# Somatostatin Receptor Expression of Gastroenteropancreatic Neuroendocrine Tumors: A Comprehensive Analysis in the Era of Somatostatin Receptor PET Imaging

**DOI:** 10.3390/cancers17121937

**Published:** 2025-06-11

**Authors:** Maria Grazia Maratta, Taymeyah Al-Toubah, Jaime Montilla-Soler, Eleonora Pelle, Mintallah Haider, Ghassan El-Haddad, Jonathan Strosberg

**Affiliations:** 1 Comprehensive Cancer Center, Fondazione Policlinico Universitario Agostino Gemelli IRCCS, Università Cattolica del Sacro Cuore, 00168 Rome, Italy; 2 Department of Gastrointestinal Oncology, H. Lee Moffitt Cancer Center & Research Institute, Tampa, FL 33612, USA; taymeyah.al-toubah@moffitt.org (T.A.-T.); eleonora.pelle@moffitt.org (E.P.); mintallah.haider@moffitt.org (M.H.); jonathan.strosberg@moffitt.org (J.S.); 3 Department of Diagnostic Imaging and Nuclear Medicine, H. Lee Moffitt Cancer Center & Research Institute, Tampa, FL 33612, USA; jaime.montillasoler@moffitt.org (J.M.-S.); ghassan.elhaddad@moffitt.org (G.E.-H.)

**Keywords:** neuroendocrine tumor, Gastroenteropancreatic NET, ^68^Ga-DOTATATE, ^64^Cu-DOTATATE, somatostatin receptor

## Abstract

**Background:** There is limited data on somatostatin receptor (SSTR) expression of metastatic gastroenteropancreatic neuroendocrine tumors (GEP-NETs) using modern imaging techniques and stratifying by primary site and tumor grade (G). Understanding patterns of SSTR expression and tumor heterogeneity is essential when determining the relevance of cold and radiolabeled somatostatin analog treatments. **Methods:** A single-institutional retrospective analysis of metastatic well-differentiated G1-3 GEP-NET patients who underwent Gallium-68 ([^68^Ga])-DOTATATE or Copper-64 ([^64^Cu])-DOTATATE positron emission tomography (PET) imaging from September 2016 to June 2024 was performed. **Results:** A total of 1192 patients were considered eligible for this study. Among them, 26 (2.2%) were completely negative on SSTR PET/computed tomography (CT), and 27 (2.3%) had weak uptake (less or equal to the normal liver). Up to 40 (3.4%) had heterogeneous SSTR expression on PET/CT: 26 (2.2%) displayed the coexistence of strongly avid lesions with the absence or near absence of SSTR uptake in measurable tumors (heterogeneous strong), while 14 (1.2%) had a combination of absent and weakly expressing SSTR tumors (heterogeneous low). An additional nine cases with prior homogeneous expression (0.8%) developed new SSTR-negative tumors along with disease progression, potentially indicating dedifferentiation. The absent or heterogeneous SSTR expression rates were greater in NET G3 than G1/G2 and in tumors originating outside the small bowel (midgut). Most NETs with absent or heterogeneous SSTR expression were fluorodeoxyglucose-F-18 ([^18^F]FDG)-avid. **Conclusions:** The large majority of metastatic GEP-NETs demonstrate strong and relatively uniform SSTR expression, but approximately 8% are SSTR-negative, weak or heterogeneous on PET/CT. Higher than average rates of absent/heterogeneous/weak SSTR expression occur in G3 NETs and lower rates among small intestine primaries.

## Simple Summary

We performed a retrospective study of 1192 patients with well-differentiated metastatic gastroenteropancreatic neuroendocrine tumors (GEP-NETs) who underwent somatostatin receptor (SSTR)-based PET/CT imaging with Gallium-68 ([^68^Ga])-DOTATATE or Copper-64 ([^64^Cu])-DOTATATE. Our findings confirmed that the large majority (approximately 92%) of metastatic GEP-NETs displayed strong, uniform SSTR expression. Only about 8% of patients had absent, weak, or heterogeneous SSTR expression. These findings were more common in grade 3 (G3) tumors and in non-midgut primary sites. Most NETs with absent or heterogeneous SSTR expression were fluorodeoxyglucose-F-18 ([^18^F]FDG) avid.

## 1. Introduction

Gastroenteropancreatic neuroendocrine tumors (GEP-NET) are characterized by an increased expression of somatostatin receptors (SSTRs) on cancer cells, particularly the SSTR2 and SSTR5 subtypes [1,2]. This distinctive feature is paramount in clinical management: SSTR expression has been used as a target for radiolabeled compounds in diagnosis and staging by SSTR-agonist scintigraphy or positron emission tomography (PET)/computed tomography (CT). In recent years, Gallium-68 DOTATATE or DOTATOC ([^68^Ga]Ga-DOTATATE or [^68^Ga]Ga-DOTATOC), or Copper-64 DOTATATE ([^64^Cu]Cu-DOTATATE) PET/CT imaging has supplanted Indium-111 ([^111^In]In)-Pentetreotide scintigraphy (OctreoScan) for whole-body staging and assessment of SSTR expression. Further, SSTR-binding drugs are commonly employed in GEP-NET therapy. All cold somatostatin analogs (SSAs), octreotide and lanreotide [3,4], and radiolabeled SSA peptide receptor radionuclide therapy (PRRT) with Lutetium-177 DOTATATE ([^177^Lu]Lu-DOTATATE) [5] are approved for advanced NET treatment, and novel experimental alpha-emitters including Lead-212 DOTAMTATE ([^212^Pb]Pb-DOTAMTATE) [6], and Actinium-225 DOTATATE ([^225^Ac]Ac-DOTATATE) [7] are being tested in clinical trials.

Understanding patterns of SSTR expression is essential when determining the relevance of SSTR-based therapies for a particular patient population. Indeed, a key criterion for radiolabeled SSA treatment is strong SSTR expression in all measurable tumors. [^68^Ga] or [^64^Cu] PET/CT scans are a key element of well-differentiated GEP-NET diagnosis, staging, and follow-up, as recommended by guidelines [8,9,10]. However, there are minimal data on SSTR expression in metastatic GEP-NET using modern imaging techniques. Older studies evaluating OctreoScans were generally limited to crude assessments of positivity or negativity and were constrained by the relatively poor sensitivity of the scan for assessment of uptake in small tumors. For example, in the phase III PROMID trial evaluating octreotide LAR versus placebo in a population of metastatic midgut NETs, 11.8% of OctreoScans were reported as “negative” [3]. A retrospective analysis by Refardt et al. on G1-2 well-differentiated NET reported 23.7% (*n* = 77/325) SSTR negative cases, which correlated with inferior survival outcomes [11]. Several studies have reported SSTR immunohistochemical (IHC) staining on biopsy or surgical tissue [12,13]. However, pathology assessment of a single sample may be affected by intra- and inter-tumor heterogeneity, thus limiting its diagnostic and prognostic value. Any assumption on the entire tumor burden, especially for metastatic and progressive diseases, could be misleading [14,15,16].

Assessments of SSTR expression in general GEP-NET populations using PET/CT are particularly limited. In a case series from China evaluating 100 GEP neuroendocrine neoplasms, including metastatic, non-metastatic, and poorly differentiated neoplasms, a positive ^68^Ga-DOTATATE PET/CT was detected in 89% of G1 GEP-NET (*n* = 8/9), 81% of G2 NET (*n* = 51/63), and only 57% of G3 NET (*n* = 8/14) [17]. Similarly, two small series on G3 GEP-NET reported an increased rate of heterogeneous/negative SSTR expression in this subgroup [18,19]. A recent study of SSTR-PET/CT imaging in lung NETs revealed a relatively high rate of SSTR-negative or heterogeneous expression patterns [20]. To our knowledge, no sizeable comprehensive study has focused on SSTR expression imaging patterns (including rates of heterogeneous expression) in metastatic well-differentiated GEP-NET of all grades.

## 2. Methods

**Figure 1:**
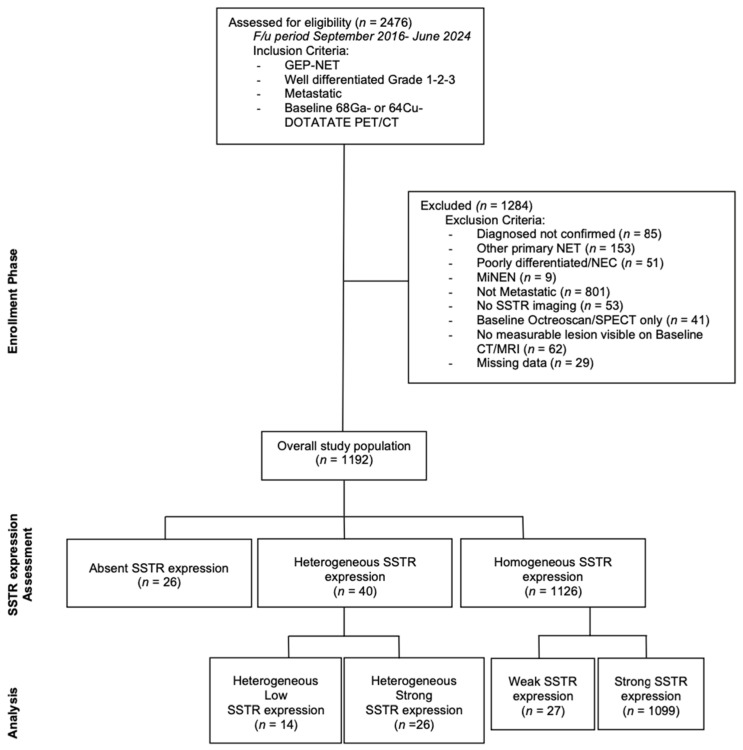
Study flowchart. GEP-NET = Gastroenteropancreatic Neuroendocrine Tumor; ^68^Ga = Gallium-68; ^64^Cu = Cupper-64; PET/CT = Positron Emission Tomography/Computed Tomography; NEC = Neuroendocrine Carcinoma, MiNEN = Mixed Neuroendocrine Non-Neuroendocrine Neoplasms; SSTR = somatostatin receptor; SPECT = Single Photon Emission Computed Tomography; CT = Computed Tomography; MRI = Magnetic Resonance Imaging.

The charts of all histologically proven GEP-NET referred to Moffitt Cancer Center (MCC) since the time of approval of [^68^Ga]Ga-DOTATATE PET/CT in the United States was reviewed. All metastatic, well-differentiated, G1-3 GEP-NET patients who underwent at least one [^68^Ga]Ga-DOTATATE or [^64^Cu]Cu-DOTATATE PET imaging at baseline between September 2016 and June 2024 were screened for the study. Among patients with unknown primary, immunohistochemical expression of Caudal-type homeobox transcription factor 2 (CDX2) on biopsy specimens was considered evidence of gastrointestinal origin. Only patients with radiographically measurable disease burden on anatomic imaging were included in the analysis. The study design is charted in Figure 1.

The standard MCC institutional imaging protocol consisted of a [^68^Ga]Ga-DOTATATE injection of 1998 kBq/kg (≤199,800 kBq) or a [^64^Cu]Cu-DOTATATE injection of 148,000 kBq 60 min before PET/CT imaging. Acquisition time per bed position was 3 min for [^68^Ga] and 4–5 min for [^64^Cu], depending on body mass index. The matrix size was 192 × 192 for [^68^Ga] and 256 × 256 for [^64^Cu]. A low-dose CT scan from the skull apex to the mid-thigh was fused with PET imaging using a Discovery MI time-of-flight scanner (GE Healthcare). Data were processed using GE Healthcare Advanced Workspace software (https://www.gehealthcare.com/) to include multiplanar reconstructions of the CT, PET, and overlayed (fused) data for interpretation.

All [^68^Ga]Ga-DOTATATE/[^64^Cu]Cu-DOTATATE PET results were compared with the most recent anatomic imaging available, contrast-enhanced computed tomography (CT) or magnetic resonance imaging (MRI). An internal review of outside scans by a Moffitt nuclear-medicine expert was performed when needed. Maximun Tumor Standardized Uptake Value (SUV_max)_ and background liver Standardized Uptake Value (SUV_liver_) were recorded whenever available in reports.

Scans were classified into five categories by visual interpretation of the degree of SSTR tracer uptake. The Krenning score was used as a reference for the assessment method proposed in this study (Table 1). All imaging series were analyzed. Longitudinal follow-up exams were also examined. All eligible cases were reviewed twice by two different clinicians.

Clinicopathologic data were also collected, including demographics, prognostic data (primary site, histological grade), and vital status. All follow-up nuclear medicine exams were collected and reviewed to assess changes in SSTR expression over time. Data on treatments were also gathered to exclude any changes in SSTR uptake attributable to treatment effects, such as tumor ablation.

Descriptive statistics were used to present patient demographics and evaluate the frequency of SSTR expression according to the following categories: uniformly strong, strong with heterogeneity, uniformly weak, weak with heterogeneity, and absent. Variations in frequency stratified by grade and tumor primary site were documented and compared using the chi-squared test. All statistical calculations were performed using IBM SPSS Statistics 26.0^®^. Institutional Review Board approval was obtained for this analysis (MCC19833/Advarra IRB Pro00030613 24OCT2018); the requirement for patient consent was waived due to the study’s retrospective nature.

## 3. Results

A total of 1192 patients were considered eligible for this study, 655 men and 537 women (M:F = 1.2:1), with a median age at diagnosis of 60 years (range 15–89). Clinicopathologic characteristics are summarized in Table 2.

**Figure 2:**
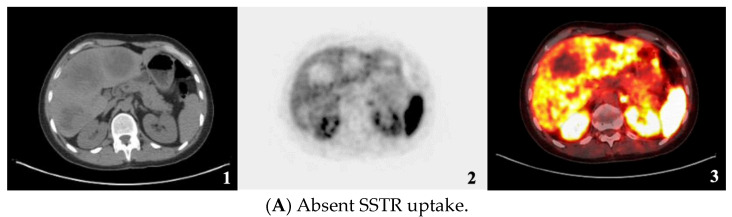

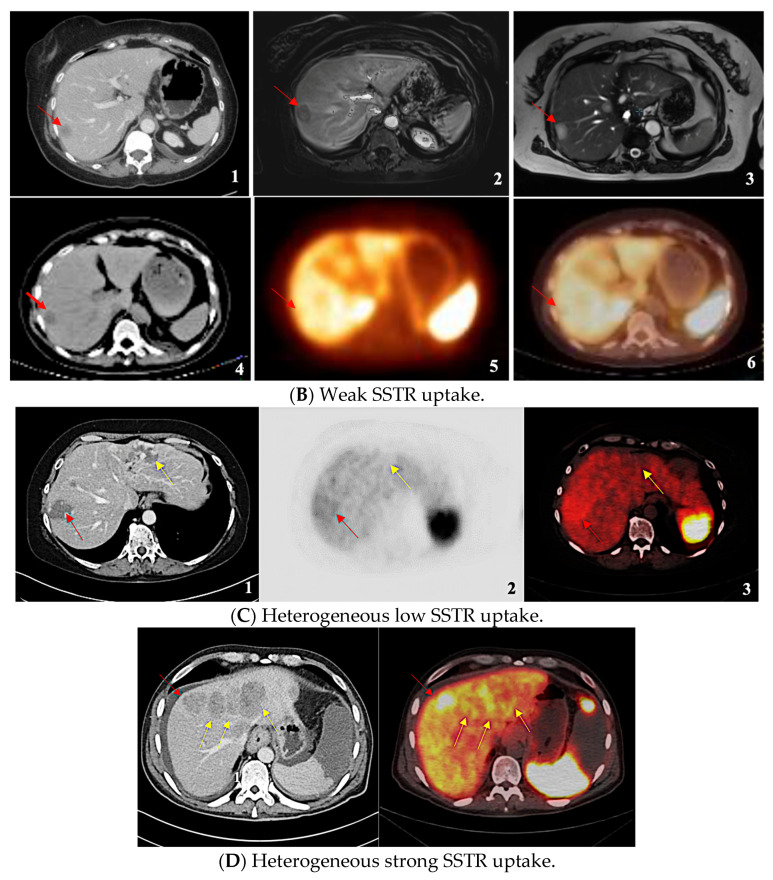
Different SSTR uptake patterns in study population: PET/CT and CT/MRI image comparison. Part (**A**) shows three SSTR negative metastatic liver lesions by different imaging methods: (1) basal CT scan acquired during [^68^Ga]Ga-DOTATATE PET/CT (axial); (2) [^68^Ga]Ga-DOTATATE PET/CT (axial); (3) Fused imaging [^68^Ga]Ga-DOTATATE PET/CT (axial). Part (**B**) shows one SSTR weak uptake metastatic liver lesion by different imaging methods (red arrow): (1) abdomen CT scan (axial, venous phase); (2) abdomen CE MRI (axial, T1); (3) abdomen CE MRI (axial, T2); (4) basal CT scan acquired during [^68^Ga]Ga-DOTATATE PET/CT (axial); (5) [^68^Ga]Ga-DOTATATE PET (axial); (6) fused imaging [^68^Ga]Ga-DOTATATE PET/CT (axial). Part (**C**) shows the coexistence of one SSTR weak and one SSTR absent uptake metastatic liver lesions by different imaging methods (red arrow for the weak lesion, yellow arrow for the absent): (1) CE CT scan (axial, venous phase); (2) [^68^Ga]Ga-DOTATATE PET/CT (axial, brightness and contrast have been edited by authors to better show metastatic lesions); (3) fused imaging [^68^Ga]Ga-DOTATATE PET/CT. Part (**D**) shows the coexistence of SSTR strong and SSTR absent/weak uptake metastatic liver lesions by different imaging methods (red arrows for the strong uptake liver lesion, yellow arrows for the absent/weak uptake liver metastasis): (1) CE CT scan (axial, venous phase); (2) fused imaging [^68^Ga]Ga-DOTATATE PET/CT (axial). SSTR = somatostatin receptor; PET/CT = Positron Emission Tomography/Computed Tomography; CT = Computed Tomography; MRI = Magnetic Resonance Imaging; ^68^Ga = Gallium-68; ^64^Cu = Cupper-64; CE = Contrast Enhanced.

Among these patients, 26 (2.2%) had completely negative SSTR expression, 27 (2.3%) had weak expression (less or equal to the normal liver), and 40 (3.4%) had heterogeneous expression. The remaining 1099 patients (92.2%) displayed strong, uniform SSTR expression. The heterogeneous disease group was further divided into two subgroups. Those with a combination of absent and weakly expressing SSTR tumors were classified as “heterogeneous low” (HL); this subgroup comprised 14 cases (1.2% of all population, 35% of the heterogeneous disease). Likewise, 26 cases displaying the coexistence of strongly avid lesions with the absence or near absence of SSTR uptake in measurable tumors were named “heterogeneous strong” (HS) (2.2% of all patients, 65% of the heterogeneous). Examples of the different patterns of SSTR expression are shown in Figure 2. Thus, in total, 7.9% of patients were either SSTR-negative or had weak or heterogeneous expression at baseline SSTR PET/CT. Results divided for employed radiotracer are reported in Table 3.

The rates of absent, weak, or heterogeneous expressive disease were greater in the NET G3 subgroup (*p* < 0.001), while there were no meaningful differences between G1 and G2. 95.1% of G1 tumors expressed SSTR uniformly and strongly compared to 92.3% of G2 tumors and only 73.7% of G3 GEP-NETs (Table 4). Rates of heterogeneous expression varied significantly by grade: 16.8% in G3 patients versus 1% for G1 patients. Remarkable differences were also seen when comparing the two most common primary sites: the small intestine and pancreas (*p* < 0.001). Less than 1% of small-intestinal NETs had absent SSTR expression compared to 4.6% of pancreatic NETs (Table 5). A total of 7.2% of pancreatic NETs had heterogeneous SSTR expression versus 1.8% of small-intestinal NETs.

A total of 15 cases changed pattern following disease progression, developing new lesions with different characteristics: three patients with absent and heterogeneous low SSTR expression showed new avid lesions (new bone, lymph node and liver metastases, respectively), while in 12 cases, patients developed new SSTR-negative tumors, potentially indicating tumor dedifferentiation. Nine of these 15 patients had a homogeneous disease at baseline (0.8%).

Data on the tumor SUV_max_ and the background SUV_liver_ were available in 874 PET/CT reports among the SSTR+ cases. In 815 of them (93.2%), the SUV_max_ was at least double that of the normal liver; in 20 (2.3%), SUV_max_ exceeded but was less than double the normal liver uptake, while in 39 (4.5%) cases (all included in the weak or HL uptake subgroups) expression was equal or below the liver background. Mean SUV_max_ values for each group are reported in Table 6.

A total of 237 patients also underwent [^18^F]FDG PET/CT evaluation. We compared the [^18^F]FDG PET/CT results with the [^68^Ga]Ga-DOTATATE or [^64^Cu]Cu-DOTATATE PET/CT results. In nine cases, the [^18^F]FDG PET/CT report showed uptake due to other concomitant diseases. Otherwise, 75% of SSTR heterogeneous and 90.9% of SSTR absent cases showed [^18^F]FDG PET/CT uptake. 71.3% of patients homogenously expressing SSTR had evidence of uptake with [^18^F]FDG as well.

## 4. Discussion

Despite the widespread use of SSTR imaging for evaluating patients with GEP-NETs, there is very little published information about expression rates in different GEP-NET subgroups and minimal data evaluating heterogeneity of SSTR expression. Descriptions of SSTR imaging as ‘positive’ or ‘negative’ are often insufficient due to phenotypic differences between cancer cells reflecting genetic and epigenetic changes that may occur under the selective pressure of microenvironment and treatment.

Our analysis of an extensive institutional database reveals that the large majority (92.2%) of GEP-NET patients express SSTR relatively strongly and uniformly. These patients are appropriate candidates for radiolabeled SSA treatment. The remaining 8% either do not express SSTR or express it weakly or heterogeneously. The percentage of patients lacking strong, uniform expression is meager in G1 and G2 GEP-NETs and is substantially higher in patients with G3 tumors. Primary site is also an important factor: small-intestine NETs tend to express SSTR strongly and uniformly at a higher rate than pancreatic NETs or tumors originating elsewhere in the gastrointestinal tract. Indeed, the rate of SSTR-negative disease in small intestinal NETs is <1%, a much lower figure than suggested by the PROMID trial in which OctreoScans were used to assess SSTR expression, likely reflecting the much higher sensitivity of PET/CT imaging.

Our study demonstrates an 8% rate of suboptimal SSTR expression in GEP-NET patients overall. An additional nine cases (0.8%) changed pattern along with disease progression, expressing heterogeneity later in their disease. Although pathology has not been documented for all cases, this evidence suggests the possibility of grade progression in GEP-NETs correlating with a decline in SSTR expression. Most GEP-NET patients without uniform SSTR expression were avid at [^18^F]FDG PET/CT in our study: 75% of SSTR heterogeneous GEP-NET and almost 90.9% of the SSTR negative group, respectively. Previously, several other studies demonstrated that [^18^F]FDG PET is complementary in SSTR negative/low expressing GEP-NETs; [^18^F]FDG PET detects higher grade, poorly differentiated disease and correlates with a worse prognosis [21,22,23,24,25]. A further step in understanding the role of a dual-tracer evaluation, including [^18^F]FDG PET, was the introduction of the NETPET score proposed by Chan et al. [26]. This new prognostic classification is based on five different grades of concordance (P1-P5) between [^68^Ga]Ga-DOTATATE and [^18^F]FDG PET uptake and has been demonstrated to correlate with overall survival in GEP-NETs [27,28] and lung NETs [29].

Our findings further support the importance of incorporating SSTR PET imaging findings into treatment planning. To assess SSTR expression heterogeneity is essential when considering cold and radiolabeled somatostatin analog therapy for metastatic GEP-NETs. Indeed, a key criterion for radiolabeled SSA treatment is strong SSTR expression in all measurable tumors. Patients with weak or heterogeneous expression may benefit more from alternative therapeutic strategies to improve patients’ outcome [30,31], particularly in cases of tumor dedifferentiation.

Limitations of this study include the possibility of false ‘positive’ findings (absent/weak SSTR lesions representing non-NET lesions rather than weak expression in NETs) and false ‘negative’ findings (inability to detect an SSTR-negative tumor in the midst of SSTR-positive metastases or due to technical factors, such as small tumor size, motion artifacts). In few ambiguous cases, we generally considered SSTR-absent/weak lesions to be likely non-NET. The interval between FDG PET and SSTR PET widely varied, accordingly to various factors including grading but also changing in disease behavior and clinician’s indication. This reflects the real word scenario and an intrinsic limit related to the retrospective nature of our analysis.

## 5. Conclusions

Most metastatic GEP-NETs express SSTR strongly and uniformly, but approximately 8% are either SSTR-negative or have weak or heterogeneous expression. Higher than average rates of absent/heterogeneous/weak SSTR expression occur in G3 NETs and very low rates among small-intestine primaries. Patterns of expression can change over time in rare cases, likely due to dedifferentiation of tumor clones. Clinicians should recognize the possibility of heterogeneity in SSTR expression, particularly in G3 tumors and non-small bowel primaries, when considering radiolabeled SSA therapy.

## Figures and Tables

**Table 1:** Proposed method for assessing the degree of tracer uptake.

SSTR Expression Assessment per Study	Krenning Score	Definition
Negative/Absent	0	No uptake
Homogeneous Weak	1	Much lower than liver
	2	Slightly less than or equal to liver
Homogeneous Strong	3	Greater than liver
	4	Greater than spleen
Heterogeneous Low	-	Mixture of absent and less than or equal to liver uptake lesions
Heterogeneous Strong	-	Mixture of strongly avid lesions combined with the absence or near absence of SSTR expression in at least one measurable tumor (primary or metastasis).

SSTR = somatostatin receptor.

**Table 2:** Clinicopathological characteristics of study population.

Parameter	*N* (=1192)	%
Sex		
Female	537	45.10%
Male	655	54.90%
Grade		
1	389	32.60%
2	613	51.40%
3	95	8%
Unknown	95	8%
Ki-67		
≤2%	389	32.60%
3–20%	581	48.70%
>20%	95	8%
Unknown	127	10.70%
Primary		
Small Bowel	784	65.80%
Pancreas	302	25.30%
Gastric	20	1.70%
Biliary-Tract	8	0.70%
Appendix	3	0.20%
Colorectal	24	2%
Unknown	51	4.30%
Hormone syndrome		
Yes	415	34.80%
No	777	65.20%

**Table 3:** Results divided into patients who underwent [^68^Ga]- or [^64^Cu]DOTATATE PET/CT.

SSTR Expression Assessment per Study	Total *N* = 1192 (100%)	[68Ga]-DOTATATE PET/CT *N* (%)	[64Cu]DOTATATE PET/CT *N* (%)
Negative/Absent	26 (2.2%)	20 (1.7%)	6 (0.5%)
Homogeneous Weak	27 (2.3%)	26 (2.2%)	1 (0.1%)
Homogeneous Strong	1099 (92.2%)	896 (75.2%)	203 (17.0%)
Heterogeneous Low	14 (1.2%)	12 (1.0%)	2 (0.2%)
Heterogeneous Strong	26(2.2%)	20 1.7(%)	6 (0.5%)

SSTR = somatostatin receptor; *N* = number; PET/TC = positron emission tomography-computed tomography scan.

**Table 4:** Breakdown of SSTR expression in each tumor grade.

SSTR Expression	G1	G2	G3	N/A
Absent	3 (0.8%)	15 (2.4%)	7 (7.4%)	1 (1.1%)
Uniformly present low expression (<liver)	12 (3.1%)	13 (2.1%)	2 (2.1%)	-
Uniformly present high expression (>liver)	370 (95.1%)	566 (92.3%)	70 (73.7%)	93 (97.9%)
Heterogeneous low expression (mixture of low and absent expression)	-	4 (0.7%)	10 (10.5%)	-
Heterogeneous strong expression (mixture of high and absent expression)	4 (1.0%)	15 (2.4%)	6 (6.3%)	1 (1.1%)
Total (100%)	389	613	95	95

SSTR = somatostatin receptor; G = grade; N/A = not available.

**Table 5:** Breakdown of SSTR expression according to primary tumor origin.

SSTR Expression	SI-NET	Pan-NET	Other
Absent	5 (0.6%)	14 (4.6%)	7 (6.6%)
Uniformly present low expression (<liver)	17 (2.2%)	7 (2.3%)	3 (2.8%)
Uniformly present high expression (>liver)	748 (95.4%)	259 (85.8%)	92 (86.8%)
Heterogeneous low expression (mixture of low and absent expression)	2 (0.3%)	8 (2.6%)	4 (3.8%)
Heterogeneous strong expression (mixture of high and absent expression)	12 (1.5%)	14 (4.6%)	-
Total (100%)	784	302	106

SSTR = somatostatin receptor; SI-NET = small intestinal neuroendocrine tumors; Pan-NET = pancreatic neuroendocrine tumors; Other = other neuroendocrine primaries.

**Table 6:** Definition and mean SUV_max_ values for analyzed groups.

SSTR Expression Assessment per Study	Krenning Score	Definition	Mean SUV_max_
Negative/Absent	0	No uptake	-
Homogeneous Weak	1	Much lower than liver	9
	2	Slightly less than or equal to liver	
Homogeneous Strong	3	Greater than liver	31.2
	4	Greater than spleen	
Heterogeneous Low	-	Mixture of absent and less than or equal to liver uptake lesions	9.5
Heterogeneous Strong	-	Mixture of strongly avid lesions combined with the absence or near absence of SSTR expression in at least one measurable tumor (primary or metastasis).	42.4

SSTR = somatostatin receptor; SUV_max_ = maximum standardized uptake value.

## Data Availability

The authors confirm that the data supporting the findings of this study are available within the article.
